# Molecular subtype analysis determines the association of advanced breast cancer in Egypt with favorable biology

**DOI:** 10.1186/1472-6874-11-44

**Published:** 2011-09-30

**Authors:** Bodour Salhia, Coya Tapia, Elia A Ishak, Salwa Gaber, Bree Berghuis, Khateeb H Hussain, Rachelle A DuQuette, James Resau, John Carpten

**Affiliations:** 1 Integrated Cancer Genomics Division, Translational Genomics Research Institute, 445 N Fifth Street, Phoenix, 85004 Arizona, USA; 2 Institute for Pathology, Basel, Switzerland; 3 Institute for Pathology, University of Berne, Berne, Switzerland; 4 Faculty of Medicine, Cairo University, Cairo, Egypt; 5 Faculty of Medicine, Minia University, Minia, Egypt; 6 Laboratory of Analytical, Cellular and Molecular Microscopy, The Van Andel Research Institute, 333 Bostwick Ave, Grand Rapids, 49503 Michigan, USA

**Keywords:** Egypt, Breast Cancer subtypes, ethnicity, early detection, Africa

## Abstract

**Background:**

Prognostic markers and molecular breast cancer subtypes reflect underlying biological tumor behavior and are important for patient management. Compared to Western countries, women in North Africa are less likely to be prognosticated and treated based on well-characterized markers such as the estrogen receptor (ER), progesterone receptor (PR) and Her2. We conducted this study to determine the prevalence of breast cancer molecular subtypes in the North African country of Egypt as a measure of underlying biological characteristics driving tumor manifestations.

**Methods:**

To determine molecular subtypes we characterized over 200 tumor specimens obtained from Egypt by performing ER, PR, Her2, CK5/6, EGFR and Ki67 immunohistochemistry.

**Results:**

Our study demonstrated that the Luminal A subtype, associated with favorable prognosis, was found in nearly 45% of cases examined. However, the basal-like subtype, associated with poor prognosis, was found in 11% of cases. These findings are in sharp contrast to other parts of Africa in which the basal-like subtype is over-represented.

**Conclusions:**

Egyptians appear to have favorable underlying biology, albeit having advanced disease at diagnosis. These data suggest that Egyptians would largely profit from early detection of their disease. Intervention at the public health level, including education on the benefits of early detection is necessary and would likely have tremendous impact on breast cancer outcome in Egypt.

## Background

The World Health Organization has ranked breast cancer as the most common type of cancer among women world-wide [1]. The incidence rates of breast cancer vary worldwide, with higher rates in North America, Northern and Western Europe; intermediate rates in South America and Southern Europe; and lower rates in Africa and Asia. According to GLOBOCAN 2008 [1] breast cancer accounts for 38% of all new cancer cases among women living in Egypt. The age-standardized rate (ASR) for breast cancer incidence in Egypt is 37.3 compared to 76 in the United States. Although incidence remains significantly lower than in highly developed countries, rates are steadily increasing [2,3]. Mortality rates in Egypt are worse (20.1 per 100 000) then they are in the United States (14.7 per 100 000), and is the leading cause of cancer-related deaths.

There is evidence that the global burden of cancer is shifting gradually to the developing world, and may equally affect or even surpass that of Western societies within the foreseeable future [1]. Despite this shift, breast cancer research in Africa comprises less than 1% of the literature as indexed in the National Library of Medicine database (PUBMED). The bias that little can be done to affect the course of third world women disease, the lack of objective measureable data and the absence of evidence-based medicine thwart the ability of clinicians to effectively manage cancer and guide health policies in Africa.

Breast cancer is a heterogeneous disease composed of a number of recognizable histological and intrinsic molecular subtypes [4-6]. The molecular subtypes, based on gene expression profiles, reflect underlying biological behavior of tumors and differ markedly in prognosis [4-6]. Intrinsic subtypes include two main subtypes: Estrogen-receptor (ER) positive (+) tumors (termed Luminal A and B) and ER negative (-) tumors (basal-like and human epidermal growth factor receptor-2-positive (Her2+) [4-6]. Luminal A and Luminal B subtypes are known to be associated with better prognostic features including low proliferative index, low histologic grade, and a tendency towards smaller tumors. However, compared to Luminal A tumors, Luminal B tumors are reported to have higher proliferation and poorer prognosis [7]. The basal-like subtype has been associated with the worst prognosis, clinical characteristics and amenability to available treatment options [5]. Molecular classifications are based on expression data from fresh-frozen samples, which are not normally prepared in third world countries. Therefore, surrogate markers based on protein expression in formalin-fixed, paraffin-embedded (FFPE) tissues are used that can predict the underlying molecular basis of breast cancer; along with the consequential prognosis and therapeutic choices of clinicians.

Currently little is still known regarding the molecular characteristics contributing to the significantly high mortality rates in regions like North Africa, including Egypt. The objective of the current study was to determine the prevalence of breast tumor molecular subtypes in an Egyptian population sampling to determine the underlying biological characteristics driving tumor manifestations in Egypt. In our study, we performed immunohistochemical analysis using the widely accepted surrogate markers that have been previously verified against gene expression profiles to accurately define the prevalence of intrinsic subtypes in our population cohort [8,9]. Such a survey will provide significant insight into underlying biological characteristics that may be predominating in a disparate patient population with seemingly aggressive tumor pathology and clinical progression. In light of minimal resources available in Africa, this work will guide future efforts in the region to determine whether an emphasis needs to be placed on biological variability or health disparities and socio-economic/cultural factors.

## Methods

### Sample Collection

Archival materials from 359 patients with confirmed breast cancer diagnosis were obtained from the University of Cairo, Faculty of Medicine, and the University of Minia, Faculty of Medicine in Egypt under appropriate institutional ethical review board approval. All samples were reviewed and verified by a board certified pathologist in each center (EH and SG). These samples represent a homogenous population comprising samples from urban (Cairo: n = 159) and rural (Minia: n = 200) areas. Samples were FFPE according to routine surgical pathology practices and collected between 2003 and 2008. Re-embedding of tissue was performed when necessary. All tumors were graded in Egypt according to the Nottingham criteria [10-12]. Pathologic features including histologic diagnosis, grade, tumor size, regional lymph node metastases as well as patient age and type of surgery performed were extracted from the pathology reports wherever possible. Although Nottingham criteria was used to stage tumors (low grade = G1, intermediate grade = G2 and high grade = G3), tumor size was categorized according to the American Joint Committee on Cancer T classification: T1: tumor ≤ 2.0 cm; T2: tumor > 2.0 cm, ≤5.0 cm; T3: tumor > 5.0 cm. T4 tumor category was excluded because of missing clinical data as, for example, presence of edema or extension to chest wall. Patients with at least one regional lymph node metastasis were considered lymph node positive.

### Construction of Tissue Microarray (TMA), Immunohistochemistry and Semi-quantitative analysis

To identify areas of invasive breast cancer whole sections were stained with hematoxylin and eosin and evaluated by trained pathologists (JR and CT). Four 1.5 mm cores from each FFPE tumor block were precisely arrayed into a new recipient paraffin block using a tissue micro-array system (Beecher Instruments, Silver Spring, MD).

Immunohistochemistry (IHC) for ER, PR, Her2, Ki67, EGFR and cytokeratin (CK) 5/6 was performed on sequential sections using referenced methods. Briefly, ER and PR staining was done with pre-diluted monoclonal mouse antibodies (DAKO K1904, Carpentaria, CA) after antigen retrieval with citrate buffer (pH 6) for approximately 45 minutes (cat# 760-107, Ventana, Tucson Arizona). Detection of Her2 and EGFR was achieved with a rabbit polyclonal anti-Her2 antibody (1:100 dilution, DAKO, cat# A0485) or a mouse monoclonal anti-EGFR antibody (prediluted, Ventana, clone 3C6) after antigen retrieval with citrate buffer as above. ER, PR, Her2 and EGFR antibodies were incubated for 32 minutes at room temperature. Antigen retrieval for Ki67 was performed with EDTA/borate/tris buffer (pH 8) for 45 minutes. Slides were incubated with a 1:50 dilution of the Ki67 antibody (Abcam, cat# 833-500) for 1 hour at room temperature. CK 5/6 staining was conducted with a monoclonal antibody (Dako, cat# IS780, 1:75) for 1 hour at room temperature after citrate buffer antigen retrieval for 45 minutes. Staining was visualized using the 3, 3-diaminobenzadine chromagen kit (Ventana, cat# 760-124) and hematoxylin counterstain. Appropriate negative controls for immunostaining were prepared by omitting the primary antibody step. Slides were prepared and stained with a Ventana Discovery XT automated immunostainer (Ventana) according to manufacturer's instructions.

The results of the immunostaining were scored semi-quantitatively by one pathologist (CT). ER and PR positivity were defined as any positive nuclear staining (i.e. ≥ 1%). Her2 was scored from 0 to 3; 0 = no staining; a score of 1 = faint, partial staining of the membrane; a score of 2 = weak complete staining of the membrane in > 10% of cancer cells; a score of 3 = intense complete staining of the membrane in > 10% of cancer cells. Her2 positive cases were defined as IHC scores greater than ≥2. CK 5/6 positivity was defined by any degree of cytoplasmatic staining in the tumor cells. EGFR was considered positive if any membranous staining was detectable. For Ki67, percent positive nuclei of tumor cells was estimated by CT. Ki67 positivity was defined as staining in greater than 1% of tumor cells.

Consistent with criteria developed from peer reviewed publications, the combinations of IHC markers used to define breast cancer molecular subtypes were as follows: Luminal A (ER positive and/or PR positive, Her2 negative, Ki67 low), Luminal B (ER positive and/or PR positive, Her2 positive and/or Her2 negative/Ki67 high), Her2+/ER- (ER negative, PR negative, Her2 positive), basal-like (ER negative, PR negative, Her2 negative, EGFR positive and/or CK5/6 positive), and unclassified (ER negative, PR negative, Her2 negative, EGFR negative and CK5/6 negative). Tumors were excluded from the study for the following reasons: missing tissue spots within the TMA, absence of invasive carcinoma, negative staining for all six markers and if scores could not be obtained for each marker being analyzed due to missing spots on a sequential array.

### Statistical Analysis

Patient and tumor characteristics were compared across breast cancer subtypes using the Chi-square (χ^2^) test for categorical variables. One-way and two-way analysis of variance (ANOVA) and the Kruskal-Wallis test were used for continuous variables. All statistical analyses were performed on GraphPad Prism version 5 (La Jolla, CA).

## Results

A total of 203 cases with invasive breast cancer were ultimately included in the study. Of these, 90 (44%) were obtained from Cairo and 113 (55.6%) were obtained from Minia (Table 1). Tables 1, 2 and 3 summarize the clinic-pathological characteristics of the study cohort by five molecular subtypes. The mean age was 51.3 years for all cases, and 46.5% were older than 50 years of age. Age was evenly distributed between sites (Table 2). Breast cancer from male patients comprised 2.5% (n = 5) of samples analyzed (Table 2). Over 70% of patients had positive regional lymph node metastases and/or tumors > 2.0 cm in size (≥T2, Table 3). Tumors were either intermediate grade (82.2%) or high grade (17.1%) and virtually none were low grade (G1) tumors (Table 3). The majority of cases (75.5%) were histologically ductal carcinomas. Medullary carcinomas represented 9.0% of tumors, which is higher than generally seen in the West (Table 3). There were site-specific differences for some clinical-pathological features. In our cohort, 81% of tumors from Minia were lymph node positive compared with 61% from Cairo (p = 0.0029, Table 4). Interestingly 25.7% of tumors in Minia were categorized as T1, significantly (p < 0.001) more than seen in the Cairene population (6.1%, Table 4). There was an association of histological grade whether tumors originated from Minia or Cairo (p < 0.0001, Table 4). Thirty percent of tumors from Minia were high grade (G3) compared with only 4.1% in Cairo. About 95% of tumors from Cairo were intermediate grade (G2) compared to only 70% in Minia (Table 4). The distribution of histological type was equal between sites (data not shown).

**Table 1:** Distribution of molecular subtypes by site of origin

	Total Tissue Cases (N = 203)	Luminal A (N = 90)	Luminal B (N = 50)	HER+/ER- (N = 24)	Unclassified (N = 16)	Basal-like[CK5/6] (N = 23)	p-value
	**No. (%)**	**No. (%)**	**No. (%)**	**No. (%)**	**No. (%)**	**No. (%)**	

**Percent of Tissue Cases**	100.0%	44.3%	24.6%	11.8%	7.9%	11.3%	

**Site**			*		*	*	* < 0.0001

Cairo, Egypt	90 (44.3%)	39 (43.3%)	20 (40.0%)	10 (41.7%)	13 (81.3%)	8 (34.8%)	

Minia, Egypt	113 (55.7%)	51 (56.7%)	30 (60.0%)	14 (58.3%)	3 (18.8%)	15 (65.2%)	

Asterisk (*) represents groups with statistically significant differences.

**Table 2:** Distribution of breast cancer molecular subtypes by age and gender

	Total Tissue Cases (N = 203)	Luminal A (N = 90)	Luminal B (N = 50)	HER+/ER- (N = 24)	Unclassified (N = 16)	Basal-like[CK5/6] (N = 23)	p-value
	**No. (%)**	**No. (%)**	**No. (%)**	**No. (%)**	**No. (%)**	**No. (%)**	

**Percent of Tissue Cases**	100.0%	44.3%	24.6%	11.8%	7.9%	11.3%	

**Sex**							

Female	198 (97.5%)	89 (98.9%)	48 (96.0%)	23 (95.8%)	16 (100.0%)	2 (95.7%)	

Male	5 (2.5%)	1 (1.1%)	2 (4.0%)	1 (4.2%)	0 (0.0%)	1 (4.3%)	

**Age, years**							

< 30	5 (2.9%)	0 (0.0%)	3 (7.0%)	1 (4.5%)	0 (0.0%)	1 (5.0%)	

30-40	23 (13.4%)	11 (14.3%)	5 (11.6%)	3 (13.6%)	0 (0.0%)	4 (20.0%)	

41-50	64 (37.2%)	25 (32.5%)	22 (51.2%)	7 (31.8%)	2 (20.0%)	8 (40.0%)	

30-50	87 (50.6%)	36 (46.8%)	27 (62.8%)	10 (45.5%)	2 (20.0%)	12 (60.0%)	

> 50	80 (46.5%)	41 (53.2%)	13 (30.2%)	11 (50.0%)	8 (80.0%)	7 (35.0%)	

Not Available	31	13	7	2.00	6.00	3.00	

Mean	51.30	52.31	48.19	51.64	57.10	50.05	0.138

Median	50.00	52.00	48.00	52.50	58.00	46.50	

SD	10.78	9.66	11.50	12.20	7.56	12.79	

Mean from Cairo	53.4	55.45	48.84	53.40	57.10	49.29	

Mean from Minia	49.46	49.95	47.67	50.38		50.46	

Age Range	24 - 80	30 - 73	25 - 80	24 - 77	42 - 70	28 - 80	

Age Range from Cairo	25 - 73	35 - 73	25 - 65	38 - 72	42 - 70	28 - 70	

Age Range from Minia	24 - 80	30 - 70	25 - 80	24 - 77		40 - 80	

**Table 3:** Tumor characteristics of breast cancer molecular subtypes in Egypt

	Total Tissue Cases (N = 203)	Luminal A (N = 90)	Luminal B (N = 50)	HER+/ER- (N = 24)	Unclassified (N = 16)	Basal-like[CK5/6] (N = 23)	p-value
	**No. (%)**	**No. (%)**	**No. (%)**	**No. (%)**	**No. (%)**	**No. (%)**	

**Percent of Tissue Cases**	100.0%	44.3%	24.6%	11.8%	7.9%	11.3%	

**Lymph Nodes**					*	*	

Positive	74 (70.5%)	33 (71.7%)	21 (77.8%)	11 (78.6%)	4 (50.0%)	5 (50.0%)	< 0.0001

Negative	31 (29.5%)	13 (28.3%)	6 (22.2%)	3 (21.4%)	4 (50.0%)	5 (50.0%)	

Not Available	98	44	23	10	8	13	

**Tumor size, cm (T category)**	*					*	

≤ 2.0 (T1)	23 (15.1%)	11 (15.9%)	5 (14.7%)	5 (23.8%)	2 (15.4%)	0 (0.0%)	* 0.0002

> 2.0-≤5.0 (T2)	111 (73.0%)	51 (73.9%)	26 (76.5%)	13 (61.9%)	8 (61.5%)	13 (86.7%)	

> 5 (T3)	18 (11.9%)	7 (10.1%)	3 (8.8%)	3 (14.3%)	3 (23.1%)	2 (13.3%)	

Not Available	51	21	16	3	3	8	

**Histological grade**							

Low Grade (G1)	1 (0.7%)	1 (1.5%)	0 (0.0%)	0 (0.0%)	0 (0.0%)	0 (0.0%)	0.0443

Intermediate Grade (G2)	120 (82.2%)	51 (78.5%)	34 (85.0%)	13 (86.7%)	10 (76.9%)	12 (92.3%)	

High Grade (G3)	25 (17.1%)	13 (20.0%)	6 (15.0%)	2 (13.3%)	3 (23.1%)	1 (7.7%)	

Not available	57	25	10	9	3	10	

**Histological Type**							

Ductal	142 (75.5%)	62 (73.8%)	41 (87.2%)	15 (68.2%)	12 (75.0%)	12 (63.2%)	< 0.0001

Lobular	17 (9.0%)	11 (13.1%)	3 (6.4%)	1 (4.5%)	1 (6.3%)	1 (5.3%)	

Mixed ductal & lobular	5 (2.7%)	3 (3.6%)	0 (0.0%)	1 (4.5%)	1 (6.3%)	0 (0.0%)	

Medullary	17 (9.0%)	3 (3.6%)	2 (4.3%)	4 (18.2%)	2 (12.5%)	6 (31.6%)	

Other	7 (3.7%)	5 (6.0%)	1 (2.1%)	1 (4.5%)	0 (0.0%)	0 (0.0%)	

Not Available	15	6	3	2	0	4	

Asterisk (*) represents groups with statistically significant differences.

**Table 4:** Breast cancer characteristics by site of origin

	Cairo (N = 90)	Minia (N = 113)	p-value
	**No. (%)**	**No. (%)**	

**Age**			

Mean	53.4	49.5	

**Lymph Nodes**			

Positive	35 (61.4%)	39 (81.3%)	0.0029

Negative	22 (38.6%)	9 (18.8%)	

Not Available	33	65	

**Tumor size, cm (T category)**			

≤ 2.0 (T1)	5 (6.1%)	18 (25.7%)	0.001

> 2.0-≤5.0 (T2)	66 (80.5%)	45 (64.3%)	

> 5 (T3)	11 (13.4%)	7 (10%)	

Not Available	8	43	

**Histologic Grade**			

Low Grade (G1)	1 (1.4%)	0 (0.0%)	< 0.0001

Intermediate Grade (G2)	69 (94.5%)	51 (69.9%)	

High Grade (G3)	3 (4.1%)	22 (30.1%)	

Not Available	17	40	

**Proliferative Index (Ki67)**			

Mean	14.9	15.8	

SD	12.3	9.8	

### Prevalence of molecular subtypes

**Figure 1:**
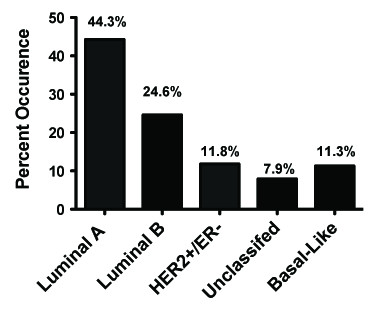
**Distribution of breast cancer molecular subtypes in Egypt**.

The 203 tumors fell into one of five molecular subgroups based on IHC criteria described above (Table 1). Overall, IHC analysis revealed that 65.0% 43.8% and 25.1% were positive for ER, PR and Her2, respectively (Table 5). We used IHC scores ≥2 to define HER2 positive tumors. Although a potential limitation of our study is the lack of HER2 FISH to accurately call Her2 positive tumors with IHC scores of 2+, our maximum false negative rate is 2% for our dataset. Furthermore, evidence is emerging that women with intermediate IHC scores of 2+ without amplification by HER2 FISH may also benefit from trastuzumab therapy [13]. The incidence of ER (p = 0.13), PR (p = 0.09) and Her2 (p = 0.51) positivity was nearly the same between Minia and Cairo (Table 5). The majority of tumors were Luminal A (44.3%), 24.6% were Luminal B and less than 12% were Her2+/ER-, unclassified or basal-like (Figure 1, Table 1). The distribution of Luminal A and Her2+/ER- subtypes was not affected by whether a sample came from Minia or Cairo. However, 60% of Luminal B tumors and 65% of basal-like tumors were from Minia, whereas 81% of unclassified cases were from Cairo (p < 0.001, Table 1). Two-way analysis of variance (ANOVA) with post hoc comparison revealed no overall association to age with different molecular subgroups (Table 2).

**Table 5:** Frequency of ER, PR and Her2 expression in Egypt

	Total Tissue Cases (N = 203)	Cairo (N = 90)	Minia (N = 113)	p-value
	**No. (%)**	**No. (%)**	**No. (%)**	

**ER**				

Positive	132 (65.0%)	58.89%	69.91%	0.138

Negative	71 (35.0%)	41.11%	30.09%	

**PR**				

Positive	89 (43.8%)	51.11%	38.05%	0.088

Negative	114 (56.2%)	48.89%	61.95%	

**Her2**				

Positive	51 (25.1%)	22.22%	27.43%	0.511

Negative	152 (74.9%)	77.78%	72.57%	

### Comparison of tumor size, histologic subtype, lymph node status and proliferation index among molecular subtypes

**Figure 2:**
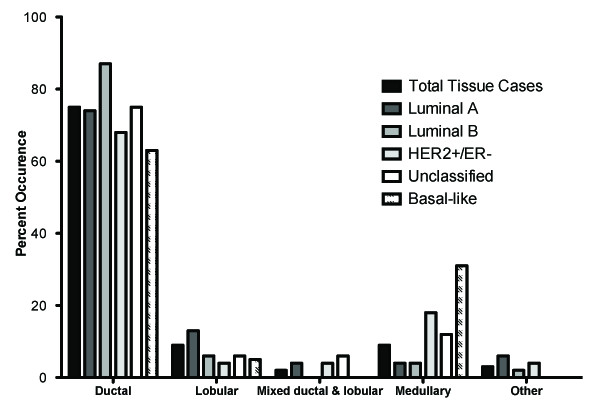
**Distribution of breast cancer histological subtypes in relation to molecular subtype**.

**Figure 3:**
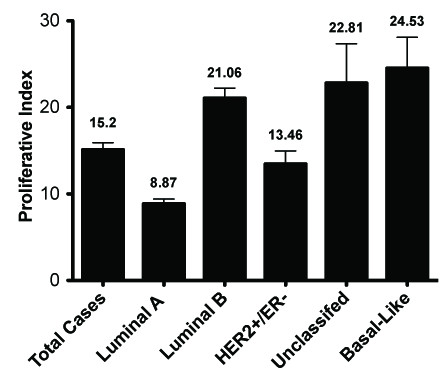
**Ki67 proliferative index of breast cancer molecular subtypes in Egypt**.

Intermediate tumor grade accounted for the majority (> 78%) of each molecular subtype and no great association of tumor grade to molecular subtype was found (Table 3). Greater than 70% of Luminal A, Luminal B, and HER2+/ER- tumors were lymph node positive (Table 3). This differed significantly from unclassified and basal-like tumors, which only had 50% lymph node positive cases (p < 0.0001). With regards to tumor size, the only noticeable distinction occurred in the basal-like group where all tumors were larger than 2.0 cm (T2 or greater) (Table 3). Tumors less than 2.0 cm represented 15-24% tumors in the other subtypes (p = 0.0002). The majority (greater than 65%) of tumors in all subtypes were of ductal origin. (Table 3, Figure 2). The basal-like group was also made up of a large number (31.6%) of medullary tumors, significantly more than in other molecular subtypes (p < 0.0001) (Table 3). Tumor proliferation was assessed for all tumors by measuring the percent positive Ki67 tumor nuclei (Figure 3). Unclassified and the basal-like tumors had the highest Ki67 scores (Figure 3). By definition, Luminal A tumors had low proliferative indices and Luminal B tumors had higher Ki67 values compared to Luminal A.

### Analysis of surgical procedure among molecular subtypes

Surgical procedures extracted from pathology reports were consolidated into four groups: biopsy, modified radical mastectomy (MRM), simple mastectomy (with or without axillary clearance) and lumpectomy (with or without axillary clearance). Modified radical mastectomy was the preferred surgical procedure and was not associated to molecular subtype. Interestingly, 89% of biopsies and 61% MRMs were performed in Minia, whereas 100% of lumpectomies were performed in Cairo (p < 0.0001, Table 6), where this was the preferred surgical approach.

**Table 6:** Types of surgery performed in Egypt

	Cairo, Egypt (N = 90)	Minia, Egypt (N = 113)	p-value
	**No. (%)**	**No. (%)**	

**Surgery**			

Biopsy	6 (6.67%)	48 (46.6%)	< 0.0001

Modified Radical Mastectomy	32 (35.56%)	51 (49.5%)	

Mastectomy ± axillary clearance	9 (10.00%)	4 (3.9%)	

Lumpectomy ± axillary clearance	43 (47.78%)	0 (0.0%)	

Not Available	0	10	

## Discussion

The Egyptian breast cancer series in our study was consistent with the expected findings of advanced disease at time of diagnosis compared to that ordinarily seen in North America and Europe. In spite of a more advanced disease at presentation, our study indicated that expression of ER, PR and Her2 resembled that of Western countries with no differences between urban and rural centers in Egypt. Furthermore, our data demonstrated that the majority of cases were classified as Luminal A (44%), which offers the best prognosis of all the subytpes.

A recent retrospective analysis of ER status in Egypt reported greater than two-fold more ER positive tumors in urban areas compared with rural areas of Egypt but that overall ER negative tumors were more prominent in the population [14]. The authors of that study suggested that higher exposure to xenoestrogens in more urbanized areas could explain the difference [14]. However, perhaps the discordance between their study and ours could also be explained by the fact that testing for ER and PR is not a routine practice in Egypt. This is witnessed from the lack of ER or PR data in over 60% of the cases examined by Dey et al (2010) [14,15].

Luminal A tumors are predominant in Asian, white and post-menopausal African North Americans. Interestingly, Luminal A was also predominant in people of Sudanese and Tunisian descent [16,17]. A high frequency of the basal-like subtype has been reported in about 40% of young premenopausal African North American women [18]; 27% in women of Nigerian and Senegalese descent [19]; 15% in post-menopausal African North American women [18]; 10% in Sudanese [17], Saudi populations [20] and in white populations [8]. The Her2 subtype has been also associated with aggressive characteristics but along with Luminal B tumors are generally not associated to ethnicity [19]. In our study, we had a significant percentage of Luminal B tumors compared to that previously reported in North American and European women (24.6% compared with 6-19%). Luminal B tumors are prognostically less favorable than Luminal A tumors but still less aggressive than basal-like tumors [10]. Until recently, most studies using IHC to assign molecular subtype status to breast cancer have not used Ki67 to discriminate between Luminal A and B and rather only used Her2 status [7]. Therefore, that we see an enrichment of Luminal B in the Egyptian population compared to other studies could also be due to the fact that other studies have simply underestimated the frequency of Luminal B cases. Unclassified tumors vary across populations and were highest among indigenous Africans and also high among 43% of breast cancer in Saudi women [19,20]. This tumor subtype is generally associated with more aggressive phenotypes [19]. In our study cohort this molecular subtype represented the smallest group.

There were some noticeable differences depending on whether tumors originated from Minia or Cairo. Minia and Cairo represent large rural and urban centers respectively. Minia with a population of 4 mllion is situated 152 miles south of Cairo on the western bank of the Nile River in Middle Egypt and represents a large rural region in Egypt. Minia is a large agricultural and industrial town and much smaller in size than one of the world's largest urban cities, Cairo with nearly 20 million inhabitants. Tumors in Minia were higher grade, and were significantly more lymph node positive but surprisingly tumors were not larger in size. In fact there was a higher frequency of tumors classified as T1 from Minia. Furthermore, surgical practices differed as well with none of the lumpectomies performed in Minia. With regards to molecular subtype, tumors from Minia represented the majority of Luminal B and basal-like tumors and also demonstrated significantly fewer unclassified cases than ones from Cairo. Patient age, histological subtype distribution, hormone receptor frequency and Her2 status did not vary by site of origin.

Although it is not entirely clear why these differences exist, collectively, they point towards socio-cultural and biological heterogeneity not accounted for between the two regions of Egypt. The differences are also likely to be an indication of inherent differences in medical practice, which may itself be related to socio-cultural factors, a dampened degree of modernity and fewer resources available in Minia. A recent study examining breast cancer in Minia found an association of poor survival with markers of poor prognosis; variable treatment profiles; lack of screening programs and treatment; lack of education; and poor literacy rates [21].

It is becoming apparent that breast cancer from women of African Arab descent differs significantly from people of Sub-Saharan Africa, suggesting important molecular epidemiological differences that need to be understood when dealing with breast cancer on the African continent. In addition, the ancestry of the Modern Egyptian could have important implications. Although this is a topic which remains controversial, two studies examined Y chromosome haplotypes in Egypt and 45 informative biallelic markers, as well as, 10 microsatellite loci on the nonrecombining region of the Y chromosome (NRY) [22,23]. The most frequent haplotype was haplotype V which is a characteristic Arab haplotype [22]. This is compared to haplotype IV which is prevalent in sub-Saharan Africa and found at a much lower frequency in Egypt [22]. Egypt's NRY frequency distributions appear to be much more similar to those of the Middle East than to any sub-Saharan African population [23]. These studies suggest a much larger Eurasian genetic component thus explaining in part the different molecular profile of breast cancer from other Sub-Saharan African nations.

The general lack of association to clinical tumor features has been reported in other studies [17,19] and could mean that intrinsic subtype is predetermined or that other ethnic-specific, not yet understood, biological mechanisms are responsible. The association between molecular subtype and tumor proliferation in our study is consistent with the literature, which indicates that basal-like and unclassified tumors have higher proliferation indices compared to Luminal A tumors [7]. Luminal B tumors by definition had higher proliferation compared with Luminal A tumors [7]. Although basal-like tumors consisted of a higher percentage of medullary tumors, studies have shown that all medullary tumors are basal-like [24]. In addition, medullary tumors were more frequently diagnosed in the Egyptian cohort (9%), whereas they comprise less than 2% of breast cancers in Western nations [25]. It is not clear why these differences exist and could be related to differences in diagnostic criteria. Whether this could also be due to a higher frequency of BRCA1 mutations, for which this histological subtype has been associated [26], diagnostic criteria or other causes still needs to be determined.

A recent study demonstrated that while African Americans had higher breast-cancer specific mortality than whites, the effect of race was only statistically significant among women with luminal A breast cancer [10]. However, mortality for patients with basal-like breast cancer was higher among whites, suggesting that basal-like breast cancer is not an inherently more aggressive disease in African American women [10]. This study is highly relevant to ours and supports the notion that poor outcomes seen in Egyptian women compared with Western women need not be explained by a higher prevalence in the basal-like subtype. Given that the distribution of molecular subtypes are similar among Arabs and Western Caucasian women, the difference in outcomes between the two populations may not have biological underpinnings. Instead, for women of Egypt, our data point towards the importance of early diagnosis, therapy received (or not received), nutrition, co-morbidity, overall general health, or potential other causes. Particularly relevant to developing and underserved communities, socioeconomic factors, availability of resources, access to care, disease awareness, and cultural stigmas are important contributing factors [15,27]. Nevertheless, performing gene expression profiling as a next step could help identify additional Luminal-associated genes unique to the Egyptian population.

## Conclusions

While the breast cancer disparity of this population is unfortunate, the results of our study are optimistic and provide an important opportunity to intervene. It is not unrealistic to conceive that "simple" and relatively inexpensive measures of increasing awareness and improving early detection alone could improve survival rates dramatically. There is ample evidence due to comprehensive clinical trials that early detection by screening along with timely and effective treatments is tightly associated with improvements in breast cancer survival in the industrialized world [16]. Factoring in measures to implement mandatory ER and PR stratification of patients with national standardized IHC protocols and proper training, whilst increasing efforts to bolster the use of hormonal therapy has the potential of matching breast cancer survival rates to that of the United States. Evidence for this comes from the fact that while minority women in the United States are more likely to present with advanced disease and have higher mortality rates than Caucasian women, white women and black women who present with similar stage of disease and receive similar treatments have similar outcomes [16].

Although there are clear and hopeful signs that programs to increase awareness and education are beginning to emerge in Egypt thanks to organizations like The Breast Cancer Foundation of Egypt, The Suzanne Mubarak Regional Centre for Women's Health and Development, The Susan G. Komen for the Cure, and the US Agency for International Development (US-AID), more is needed in the way of patient outreach, resources, and improving training for physicians throughout. This study will help lead to new and needed bio-repository efforts in Egypt and other nations in Africa, which will facilitate further research and help to determine other bio-ethnic specific variability. Finally, with such few resources available in Africa we need to understand and report biological differences in order to proceed in the most efficient and effective manner. The impact of our study doesn't stem from the identification of novel biomarkers or therapeutics, but from its utility; there is a real opportunity to help save the lives of many in Egypt by simply improving rates of early detection, and utilizing a set of already known prognostic and therapeutic markers.

## Abbreviations

ANOVA: analysis of variance; ASR: age-standardized rate; CK 5/6: cytokeratin 5/6; EDTA: Ethylenediaminetetraacetic acid; EGFR: epidermal growth factor receptor; ER: estrogen receptor; FFPE: formalin-fixed paraffin-embedded; Her2: human epidermal growth factor receptor-2-positive; IHC: immunohistochemistry; MRM: modified radical mastectomy; NRY: non-recombining region of the Y chromosome; PR: progesterone receptor; TMA: tissue microarray.

## Competing interests

The authors declare that they have no competing interests.

## Authors' contributions

BS was responsible for the study concept, study design and managed study. BS participated in data acquisition (TMA construction), data analysis, statistical analysis, and wrote manuscript. CT was the pathologist responsible for scoring ER, PR, HER2, Ki67, EGFR and CK5/6 and reviewed cases for inclusion in study. CT provided input towards study design and reviewed and edited manuscript. EAI was the pathologist diagnosing and providing all cases from Cairo. SG was the pathologist diagnosing and providing all cases from Minia. BB assisted with data acquisition, histotechnology skills (TMA sectioning and staining). KHH - Assisted with data analysis. RAD - Assisted with data analysis. JR was in charge of TMA region selection, construction and IHC staining was performed in JR's lab under direct supervision. JR provided guidance and mentorship and reviewed and edited manuscript. JC provided guidance and mentorship and edited manuscript. All authors read and approved the manuscript.

## Pre-publication history

The pre-publication history for this paper can be accessed here:

http://www.biomedcentral.com/1472-6874/11/44/prepub
